# Concerns about the ‘corporate capture’ of The Academy article

**DOI:** 10.1017/S1368980023000551

**Published:** 2023-09

**Authors:** J Lauren Butler, Leia Downs, Cassandra M Johnson, Lindsey Menge, Ramona Salcedo Price, Sandy Roberts, Christy Youens

**Affiliations:** Nutrition and Foods Program, School of Family and Consumer Sciences, Texas State University, San Marcos, TX 78666, USA

We are writing in response to a research article published online in Public Health Nutrition on 24 October 2022, about the corporate capture of The Academy of Nutrition and Dietetics (or The Academy for short)^(1)^. In their paper, Carriedo, Pinsky, Crosbie, et al. reported on an inductive analysis and triangulation of publicly available Academy documents. The article concludes that The Academy and its Foundation have a symbiotic relationship with corporations that conflicts with The Academy’s mission to improve health^(1)^. In response to Carriedo et al.’s article, The Academy published a point-by-point rebuttal that identified their concerns and described the article’s errors^(2,3)^. While The Academy published a response and rebuttal immediately^(2,3)^, the amplified platform Carriedo et al.’s paper was given generated a highly visible source of misinformation^(1)^, with negative implications for the field.

For decades, Public Health Nutrition has been considered a trusted journal by researchers, practitioners, and policymakers in the USA and around the world. In fact, this journal has published leading articles on complex and critically important issues in Public Health Nutrition such as food insecurity (exemplar articles^(4–6)^). My colleagues, both registered dietitians (RDs) and non-RDs in public health nutrition strongly disagree with the decision to publish and categorise the Carriedo et al. manuscript as a research paper. It is further concerning that the peer review and publication process did not find cause for rejection or substantial revision. This letter formally documents our collective concerns and asks the journal to take a stand against ethical power plays.

First, the authors’ affiliations with the US Right to Know non-profit organisation and connections to the Organic Consumers Association^(1)^ are noteworthy, particularly given the focus of the article on corporate relations. Moreover, as a qualitative research paper, the article does not meet minimum standards for design or research methodology foundational to qualitative inquiry. Specifically, authors were not forthcoming in describing how their lived experience, training or roles influenced this research. There is no mention or description of their worldviews or the theoretical or empirical foundations that shaped their inquiry. Their description of data collection, analysis and interpretation was inadequate to support validity and reliability. It is very likely that their findings would not be reproducible if another research team were to re-code documents, identify major and minor themes based on reading, coding, and discussion of findings, interpret themes, and report their findings. Typically, a well-designed and well-written qualitative paper provides considerable detail regarding the research process. However, in this paper, the study design and methods were not appropriate to answer the research question, and the conclusions were unsupported at best. Usually, a manuscript that has ‘fatal flaws’ would be rejected by peer reviewers.

Additionally, the paper did not acknowledge context to their rationale, nor did it consider contextual factors in the analysis, or report of results. At best, this article by Carriedo et al.^(1)^ represents a gross over-simplification of complex dynamics between professional and trade societies, their corporate stakeholders, and professionals in research, practice, and policy. Professional and trade societies must build relationships with industry and corporate partners. This is true for all professional societies not only The Academy. For example, the article’s acknowledgements section lists several donations to the US Right to Know from advocacy groups and private funders including donations totalling $1 032 500 from the Organic Consumers Association and $397 600 from the Laura and John Arnold Foundation since 2014. As the Carriedo et al. paper mentioned^(1)^, critics have identified opportunities for The Academy to acknowledge and address ethical issues. Perhaps The Academy and a named leader (Past President of The Academy) were easy targets. However, the paper failed to mention The Academy’s role in advocating for policy changes in nutrition, supporting workforce training in nutrition and dietetics, and providing critical guidance for RDs. For example, The Academy publishes the Nutrition Care Manual needed for RDs to provide evidenced-based medical nutrition therapy to hospitalised patients^(7)^. Publishing the paper by Carriedo et al. unequivocally damages the field of nutrition-related public health with unsupported claims, such as an implication that The Academy and its Foundation use professionals and students, partnerships, and policies to assist the food and beverage, pharmaceuticals, and agribusiness industries (see p. 3581 of paper^(1)^).

In summary, this article did not report on rigorous or relevant research, which is not good science; their methods and reporting did not consider context, which compromises validity; and did not present accurate or reliable information, which generates an abundance of misinformation. Based on our opinion, publishing this article was irresponsible. Per the journal’s website, the scope ‘includes multi-level determinants of dietary intake and patterns, anthropometry, food systems, and their effects on health-related outcomes^(8)^’. As a top journal, Public Health Nutrition has a responsibility to publish rigorous and relevant research that advances nutrition-related public health and minimises potential for harm. On behalf of my colleagues in Public Health Nutrition, we strongly encourage you to review this manuscript against: (1) the standards for qualitative research such as the Consolidated Criteria for Reporting Qualitative Studies (COREQ) checklist^(9)^, which is used by The Academy’s Journal^(10)^, and (2) the recommendations for researchers to lead strategic science with policy impact^(11)^. Then, please consider which actions the journal can take to stand up for science and our field. Some potential options include publishing an erratum and requiring Carriedo et al. to revise their manuscript to meet publication standards (including providing a quality checklist for a qualitative study), revoking publication, or inviting other teams of qualitative researchers to repeat this ‘study’ and publish their findings in Public Health Nutrition. Additional options the journal could take include writing a statement or position paper on the importance of balancing rigour and ethics in research, soliciting manuscripts for a scholarly debate regarding achievements, situations, and problems related to corporate relations with professional nutrition societies (as mentioned on the Public Health Nutrition website^(8)^), or another not-yet-identified action that will promote scholarly dialogue in Public Health Nutrition. Thank you for your attention to our concerns.
